# Transforming growth factor beta (TGF-β) is activated by the CtBP2-p300-AP1 transcriptional complex in chronic renal failure

**DOI:** 10.7150/ijbs.38841

**Published:** 2020-01-01

**Authors:** Ping Zhou, Xiaoxiao Wan, Yan Zou, Zhi Chen, Aimin Zhong

**Affiliations:** 1 Department of Nephrology, Jiangxi Provincial People's Hospital Affiliated to Nanchang University, Nanchang 330006, Jiangxi, China.; 2 Department of Critical Care Medicine, Jiangxi Provincial People's Hospital Affiliated to Nanchang University, Nanchang 330006, Jiangxi, China.

**Keywords:** TGF-β, CtBP2, p300, c-Jun, c-FOS, chronic renal failure

## Abstract

Chronic renal failure (CRF), also known as chronic kidney disease (CKD), is a common renal disorder characterized by gradual kidney dysfunction. Molecular dissection reveals that transforming growth factor beta (TGF-β) plays a central role in the pathogenesis of CRF. However, the mechanism underlying TGF-β upregulation has not been demonstrated. Here, we verified that the elevated level of TGF-β was associated with the severity of CRF stages and the activation of TGF-β-mediated signaling in 120 renal biopsies from CRF patients. By analyzing the promoter region of the *TGFB1* gene, we identified one AP-1 (activator protein 1) and four NF-κB (nuclear factor kappa-light-chain-enhancer of activated B cells) binding sites. Knockdown of two AP-1 subunits (*c-Jun* and *c-FOS*) or blockage of AP-1 signaling with two inhibitors T-5224 and SR11302 could cause the downregulation of *TGFB1*, whereas knockdown of two NF-κB subunits (*p65* and *p50*) or blockage of NF-κB signaling with two inhibitors TPCA1 and BOT-64 could not change the expression of *TGFB1*. Using mass spectrometry and coimmunoprecipitation analyses, we found that both c-Jun and c-FOS formed a complex with CtBP2 (C-terminal binding protein 2) and histone acetyltransferase p300. Our *in vitro* data demonstrated that induction of CtBP2 by recombinant IL-1β (interleukin-1 beta) led to the upregulation of *TGFB1* and the activation of TGF-β downstream signaling, while knockdown of *CtBP2* resulted in the reversed effects. Using chromatin immunoprecipitation assays, we revealed that the CtBP2-p300-AP1 complex specifically bound to the promoter of *TGFB* and that knockdown or blockage of CtBP2 significantly decreased the occupancies of the p300 and AP-1 subunits. Our results support a model in which the CtBP2-p300-AP1 transcriptional complex activates the expression of *TGFB1*, increasing its production and extracellular secretion. The secreted TGF-β binds to its receptors and initiates downstream signaling.

## Introduction

Chronic renal failure (CRF), also called chronic kidney disease (CKD), is a long-standing and progressive dysfunction of the kidney 1, 2. The overall prevalence of CRF in the U.S. adult population is nearly 15% 3, 4, and CRF has become a major public health issue that affects the life quality of individual patients and increases social burden 5-7. The molecular mechanisms reveal that multiple signaling pathways, such as TGF-β (transforming growth factor-β)-mediated signaling, Wnt signaling, AKT (AKT serine/threonine kinase, also known as protein kinase B) signaling and NF-κB (*nuclear factor* kappa-light-chain-enhancer of activated B cells) signaling, are activated in the pathogenesis of CRF 8-12. Of these pathways, TGF-β-mediated signaling plays a central role 8-12. Elevated levels of TGF-β have been found in CRF patients and in animal models in many laboratories 13-15. Repression of its coding gene *TGFB1* or blockage of TGF-β function using its specific antibody can decrease renal scarring 13-15. Increased TGF-β initiates its downstream signaling through binding to a type II TGF-beta receptor (TGFBR2), which recruits and phosphorylates a type I receptor (TGFBR1). TGFBR1 further phosphorylates Smad2 and Smad3, followed by forming a transcriptional complex with Smad4. The accumulations of this complex in the nucleus enable it to regulate the expression of multiple genes, such as *CTGF* (connective tissue growth factor) and *ECM* (extracellular matrix) 13-15.

Transcription factors (TFs) play fundamental roles in the regulation of gene expression 16, 17. TFs often coordinate with coactivators or corepressors and histone modification enzymes to recruit RNA polymerase II in the promoters of specific genes 16, 17. Coactivators such as CBP (CREB-binding protein) and EP300 (histone acetyltransferase p300, also known as p300) are directly recruited by TFs to gene promoters to increase gene expression 18, 19. Corepressors such as CtBPs (C-terminal binding proteins) and NCoR (nuclear receptor corepressor 1) cannot bind to TFs directly but instead interact with repressors to negatively regulate gene expression 20. Of these corepressors, CtBPs have been well investigated, and they mainly function as oncogenes to repress the expression a variety of genes such as *CDH1* (cadherin 1, also known as E-cadherin), *Bax* and *Bim* (BCL2 associated X and BCL2 interacting mediator of cell death, respectively), and *BRCA1* (breast cancer susceptibility gene 1) 20. Biochemically, CtBPs directly interact with transcriptional repressors such as HDAC1 (histone deacetylase 1) and HDAC2 or transcriptional activators such as p300 and CBP through a conserved PXDLS motif (where X represents any amino acid) 20. These repressors and activators are then recruited by TFs such as KLF3 (Kruppel-like factor 3) 21, KLF8 22, Runx2 (RUNX family transcription factor 2) 23, and TCF4 (Transcription factor 4) 24. Although CtBP1 and CtBP2 share over 80% identity in their protein sequences, they do not show significant redundancy in their functions 20. Intriguingly, in addition to their repressive functions, some studies have also revealed that CtBPs have transcriptional activation ability 25-28. For example, CtBP2 associates with KLF8 to directly activate the expression level of *TIAM1* (T-lymphoma invasion and metastasis-inducing protein 1) to promote human cancer cell migration 25. In human multidrug resistance (MDR) cancer cell lines, CtBP1 directly activates MDR1 gene expression, thereby increasing P-glycoprotein levels and drug resistance 26. In gastrointestinal endocrine cells, CtBP1 associates with RAS-responsive element binding protein 1 (RREB1), LSD1 (lysine demethylase 1) and PCAF (P300/CBP-associated factor) to activate NeuroD1-dependent transcription 27. In the somatic tissue differentiation process, ZNF750 (zinc finger protein 750) physically interacts with KLF4, RCOR1 (REST corepressor 1) and CtBP1/2 to induce the expression of epidermal differentiation genes such as *PPL* (periplakin), *PKP1* (plakophilin 1) and *DLX5* (distal-less homeobox 5) 28.

Although the elevated production of TGF-β cytokines has been observed in the process of CRF for many years 29, the underlying mechanism of its induction is still obscure. In the present study, we primarily verified the induction of TGF-β and the increase in *TGFB1* expression at the transcriptional level in kidney biopsies from CRF patients. We then focused our studies on revealing the mechanism of *TGFB1* induction *in vitro,* and our results revealed that the CtBP2-p300-AP1 transcriptional complex was responsible for the upregulation of *TGFB1*. Our results provide a potential therapeutic strategy of CRF by targeting individual members of the CtBP2-p300-AP1 complex or blocking their interactions.

## Materials and methods

### Cell line, cell culture and cell transfection

A normal human renal cell line, RPTEC/TERT1 OAT3, was purchased from the American Type Culture Collection (ATCC) (Manassas, VA, USA, #CRL-4031-OAT3). Cells were grown in Dulbecco's modified Eagle's medium (DMEM)/F12 medium (Thermo Fisher Scientific, Waltham, MA, USA, #12634010) containing 10% fetal bovine serum (FBS) (Sigma-Aldrich, Shanghai, China, #F4135) and 1% penicillin-streptomycin antibiotics (Sigma-Aldrich, #P4333) at 37 °C. Cells under 80% confluences were used for transfection to knock down or overexpress genes following a previous protocol 30. Briefly, siRNAs specifically targeting genes were purchased from Santa Cruz Biotechnology (Dallas, TX, USA), and their catalog numbers were sc-29410 (p65), sc-29407 (p50), sc-29223 (c-Jun), sc-29221 (c-FOS) and sc-37767 (CtBP2). Another *CtBP2*-siRNA was obtained from ThermoFisher Scientific (#289298). Plasmids including pCDNA3-2×Flag-c-FOS, pCDNA3-2×Flag-c-Jun, pCDNA3-6×Myc-CtBP2, pCDNA3-2×Flag-p300 and pCDNA3-6×Myc-p300 were constructed in their corresponding empty vectors. For each transfection, 50 pmol of siRNA or 1 μg of plasmid was transfected into cells using Lipofectamine 2000 (ThermoFisher Scientific, #11668019) following the manufacturer's protocol. Cells were then incubated at 37 °C for 48 h, followed by subjecting to the required experiments.

### Blood sample collection and measurement of serum TGF-β

Venous blood samples were collected from 24 healthy volunteers (control) and 120 CRF patients who represented 5 different stages (n=24 in each stage) according to their glomerular filtration rates (GFR): 1 (GFR ≥ 90 mL/min/1.73 m^2^); 2 (60 < GFR< 89 mL/min/1.73 m^2^); 3 (30 < GFR< 59 mL/min/1.73 m^2^); 4 (15 < GFR< 29 mL/min/1.73 m^2^); and 5 (GFR < 15 mL/min/1.73 m^2^) 7. All patients were diagnosed with CRF and received therapy in the Department of Nephrology, Jiangxi Provincial People's Hospital, during 2012-2016, and their basic characteristics are summarized in Supplementary Table 1. Blood samples were kept in K2EDTA-coated tubes (BD, Franklin Lakes, NJ, USA, #367835) until use. All participants signed a consent form reviewed and approved by the ethical board of Jiangxi Provincial People's Hospital in China. The serum concentration of TGF-β was determined by an ELISA kit (ThermoFisher Scientific, #BMS249-4).

### Renal biopsy collection

Percutaneous biopsies were collected from 24 renal cell adenocarcinoma patients (TNM stage 1, used as controls) and 120 CRF patients (n=24 in each stage, and these patients were the same as those for blood sample collection). All participants signed a consent form reviewed and approved by the ethical board of Jiangxi Provincial People's Hospital in China. The primary purpose of renal biopsy collection was to confirm the diagnosis of CRF using immunofluorescence (IF). All patients were given local anesthesia, and the biopsies were collected with disposable, automatic and spring-loaded devices using a 16-gauge needle. After collection, individual biopsies were split into three parts and used for RNA isolation, protein isolation and IF.

### RNA isolation and qRT-PCR assay

Renal biopsies and cultured cells were applied to isolate total RNA using the TRIzol reagent (Sigma-Aldrich, #T9424) following a protocol provided by the manufacturer. The RNA concentration was quantified using a NanoDrop, and 1.0 μg of RNA was utilized to synthesize cDNA using a ProtoScript First Strand cDNA Synthesis Kit (NEB, Beijing, China, #E6300S). The resulting cDNA was diluted 10-fold and then subjected to qRT-PCR analysis to detect gene expression using the primers listed in Supplementary Table 2. β-Actin was used as an internal control to normalize the individual gene expression level.

### Western blotting and protein level quantification

Renal biopsies and cultured cells were applied to isolate total protein extracts using 1×RIPA buffer (Sigma-Aldrich, #R0278). The protein concentration was determined using a NanoDrop, and 50 μg of protein of each sample was loaded into 10% SDS-PAGE gels, followed by transfer to PVDF membranes (Bio-Rad, Shanghai, China, #1620177). The PVDF membranes were then blocked with 5% skim milk in 1×TBST for 1 h, followed by probing with the primary antibodies including anti-Smad2 (Abcam, Shanghai, China, #ab63576), anti-pSmad2 (Sigma-Aldrich, #AB3849-I), anti-Smad3 (Abcam, #ab40854), anti-pSmad3 (Abcam, #ab52903), anti-CtBP2 (Abcam, #ab128871), anti-c-Jun (Santa Cruz Biotechnology, #sc-166540), anti-c-FOS (Santa Cruz Biotechnology, #sc-81209), anti-p300 (Sigma-Aldrich, #P2859), anti-TGF-β (Sigma-Aldrich, #SAB4502954), anti-Flag (Sigma-Aldrich, #SAB4200071), anti-Myc (Sigma-Aldrich, #05419), and anti-GAPDH (Abcam, #ab181602). After washing 5 times with 1×TBST, the membrane was probed with secondary antibodies, and the protein signals were detected using an ECL mixture (Sigma-Aldrich, #GERPN2209) and recorded by a ChemiDoc MP imaging system (Bio-Rad, #17001402).

### Chemical treatments

Cells were treated with two NF-κB inhibitors, TPCA1 (5 μM, Sigma-Aldrich, #T1452), and BOT-64 (5 μM, Santa Cruz Biotechnology, #sc-222062), and two AP-1 inhibitors, T-5224 (50 μM, GLPBIO, Montclair, CA, USA, #GC16165) and SR11302 (50 μM, Santa Cruz Biotechnology, #sc-204295), for 4 h and then stimulated with 50 ng/mL recombinant IL-1β for 2 h. To block CtBP2-mediated signaling, cells were treated with two CtBP inhibitors, MTOB (5 μM, MedKoo Biosciences, Morrisville, NC, USA, #561414) and NSC95397 (5 μM, Sigma-Aldrich, #N1786). In addition, cells were also only treated with different concentrations (0, 5, 50 and 500 ng/mL) of IL-1β. The resulting cells were applied to RNA or protein isolation to detect gene expression or protein level changes.

### Immunoprecipitation and mass spectrometry

Cells expressing pCDNA3-2×Flag-c-FOS or pCDNA3-2×Flag-c-Jun were lysed using 1× RIPA buffer containing 1×protease inhibitor (Sigma-Aldrich, #S8830), followed by centrifuging at 13,000 rpm for 15 min. The supernatant was immunoprecipitated with anti-Flag agarose (Sigma-Aldrich, #A2220). The purified Flag-c-Jun and Flag-c-FOS-associated complexes were separated on an SDS gel and stained with a Pierce Silver Stain Kit (ThermoFisher Scientific, #24612). The stained bands were excised and fragmented as described previously 30. The resulting proteins were analyzed with a Q Exactive^TM^ HF-X Hybrid Quadrupole-Orbitrap^TM^ Mass Spectrometer (ThermoFisher Scientific, #0726042). The obtained data were blasted in the NCBI database using the MASCOT search engine (V.2.3).

### Coimmunoprecipitation (Co-IP)

Cells were transfected with the following combinations of plasmids: pCDNA3-6×Myc+pCDNA3-2×Flag-c-Jun; pCDNA3-6×Myc+pCDNA3-2×Flag-c-FOS; pCDNA3-6×Myc-CtBP2+pCDNA3-2×Flag-c-Jun; pCDNA3-6×Myc-CtBP2+pCDNA3-2×Flag-c-FOS; pCDNA3-6×Myc+pCDNA3-2×Flag; pCDNA3-6×Myc+pCDNA3-2×Flag-p300; pCDNA3-6×Myc-CtBP2+pCDNA3-2×Flag; pCDNA3-6×Myc-CtBP2+pCDNA3-2×Flag-p300; pCDNA3-6×Myc+pCDNA3-2×Flag-c-Jun; pCDNA3-6×Myc+pCDNA3-2×Flag-c-FOS; pCDNA3-6×Myc-CtBP2+pCDNA3-2×Flag-c-Jun; and pCDNA3-6×Myc-CtBP2+pCDNA3-2×Flag-c-FOS. After incubation at 37 °C for another 48 h, cells were lysed using 1× RIPA buffer containing 1×protease inhibitor. Total cell lysates were centrifuged at 13,000 rpm for 15 min, and the supernatant was immunoprecipitated with anti-Flag agarose and anti-Myc-agarose (Sigma-Aldrich, #A7470) to pull down proteins. The input and output proteins were detected using western blotting analyses.

### Chromatin immunoprecipitation (ChIP) assay

Cells (5×10^6^) were washed twice with PBS buffer and incubated with 1% formaldehyde at room temperature for 15 min, followed by adding glycine to a final concentration of 125 mM for another 5 min. Cells were further rinsed twice with PBS buffer. The resulting cells were then applied to the ChIP assay using a high sensitivity kit (Abcam, #ab185913) following a protocol provided by the manufacturer. Antibodies used for immunoprecipitation included anti-CtBP2, anti-c-Jun, anti-c-FOS and mouse IgG (Santa Cruz Biotechnology, #sc-2025). The relative enrichment of promoters was determined by qRT-PCR using the primers listed in Supplementary Table 3.

### Statistical analysis

All experiments in this study were independently repeated in triplicate. Data are presented as the mean ± SEM from a representative experiment. Statistical analyses were performed using SPSS version 22 software. The significance levels were set at *P* < 0.05 (*), *P* < 0.01 (**) and *P* < 0.001 (***).

## Results

### TGF-β levels were elevated, and TGF-β-mediated signaling was activated in CRF patients

**Figure 1:**
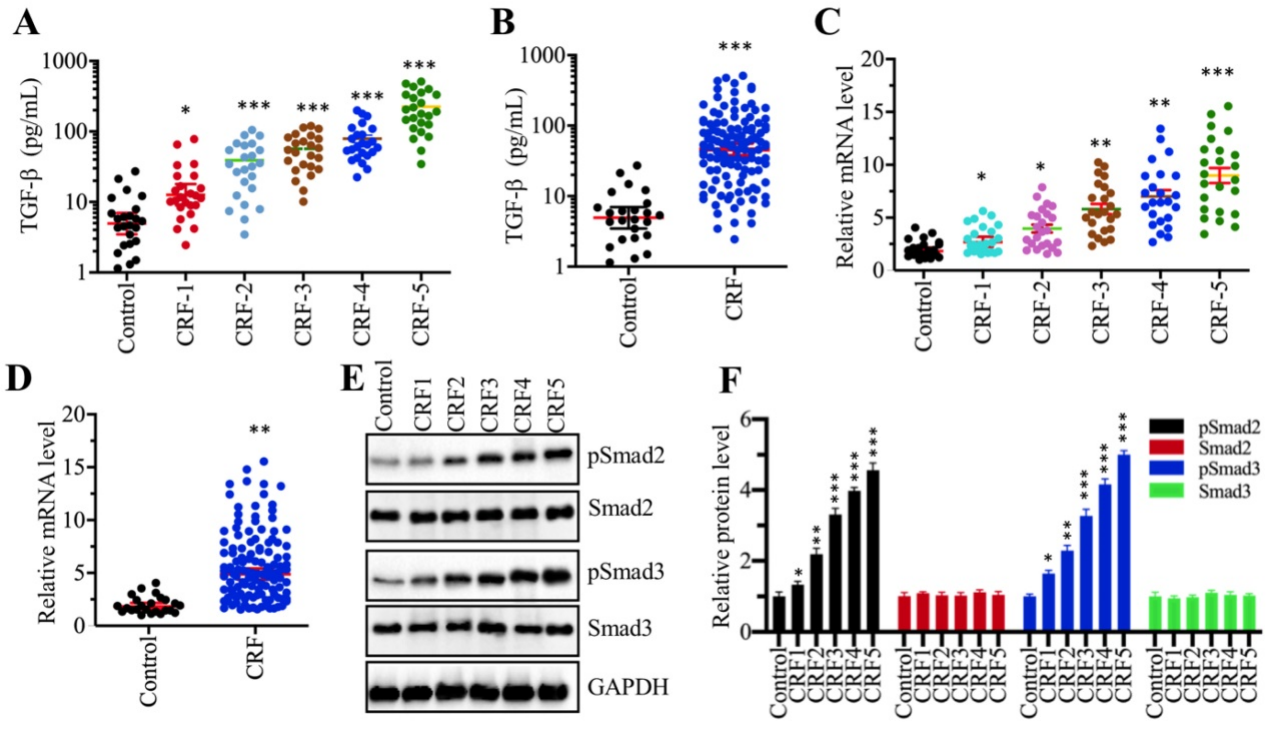
** The serum concentration of TGF-β was elevated and TGF-β signaling was activated in CRF patients (A)** Serum concentrations of TGF-β were associated with the CRF stage. Circulating levels of TGF-β were measured in serum samples obtained from healthy controls (n = 24) and CRF patients at 5 different stages (1-5, n=24 in each stage) *P < 0.05 and *** P < 0.001. **(B)** Serum concentrations of TGF-β were elevated in CRF patients. Circulating levels of TGF-β in CRF patients (n=120) were combined and compared with levels in controls. *** P < 0.001.** (C)** The relative expression level of *TGFB1* was associated with the CRF stage. The qRT-PCR analyses were performed to measure *TGFB1* mRNA levels in renal biopsies from renal cell adenocarcinoma patients (TNM stage 1, control) and 120 CRF patients representing 5 different stages (n=24 in each stage). *P < 0.05, **P < 0.01 and *** P < 0.001. **(D)** The relative expression level of *TGFB1* was elevated in CRF patients. The relative expression levels of *TGFB1* in CRF patients (n=120) were combined and compared with levels in controls. ** P < 0.01. **(E** and** F)** TGF-β signaling was activated in CRF patients. **(E)** Western blotting was performed to measure pSmad2, Smad2, pSmad3 and Smad3 in renal biopsies from one renal cell adenocarcinoma patient (TNM stage 1, control) and 5 CRF patients (n=1 in each CRF stage). GAPDH was used as a loading control. **(F)** The relative protein levels in (E) were quantified and normalized to GAPDH. *P < 0.05, **P < 0.01 and *** P < 0.001.

Although many publications have reported an increase in TGF-β levels in CRF patients 13, 29, it is still unclear whether TGF-β levels are associated with the severity of CRF stages. To verify this hypothesis, we measured circulating TGF-β levels in serum from 24 healthy volunteers and 120 CRF patients who underwent 5 different stages (from stage 1 to 5, n=24 in each stage). Our results showed that TGF-β levels in CRF patients were significantly increased compared to controls (Figure 1A). More importantly, the circulating concentration of TGF-β was positively associated with the severity of CRF stages, that is, the higher the TGF-β concentration, the more severe is the CRF (Figure 1A). Then, we combined the TGF-β concentration in individual CRF patients and compared them with healthy controls. Consistently, we also found a remarkable increase in TGF-β levels in CRF patients (Figure 1B). The increased level of TGF-β implied that its encoded gene *TGFB1* might be upregulated in CRF patients. To test this hypothesis, we isolated total RNA from renal biopsies of CRF patients and examined *TGFB1* mRNA levels in these clinical samples. Consistent with its circulating level, we also found that *TGFB1* mRNA levels were gradually increased with the severity of the CRF stages (Figure 1C). The combined data also indicated that *TGFB1* mRNA levels were dramatically increased in CRF patients (Figure 1D). It is well known that the accumulation of TGF-β can activate its downstream signaling, causing an increase in Smad2 and Smad3 phosphorylation levels. Thus, we also examined the total and phosphorylated levels of Smad2 and Smad3 in one control biopsy and 5 CRF biopsies at different stages. As shown in Figures 1E and 1F, the phosphorylated levels of Smad2 and Smad3, but not their total protein levels, were gradually increased with the severity of CRF stages. These results clearly indicated that the TGF-β level was elevated and that TGF-β-mediated signaling was activated in CRF patients.

### AP-1 specifically regulated the expression of *TGFB1 in vitro*

**Figure 2:**
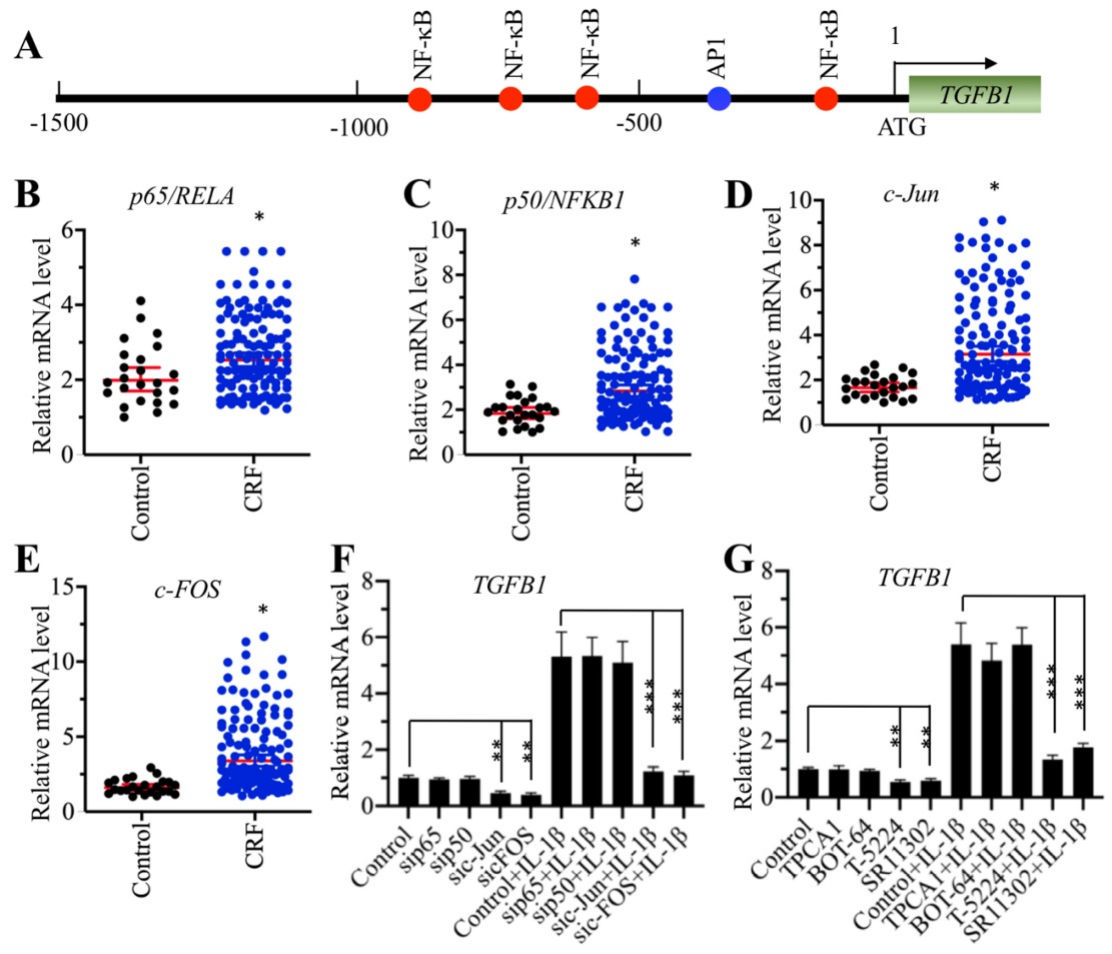
** Knockdown of AP-1 subunits caused the downregulation of *TGFB1*. (A)** The promoter of *TGFB1* contained one AP-1 and four NF-κB binding sites. A 1500 bp-length *TGFB1* promoter was selected and applied to predict transcription factor binding sites from a website (http://alggen.lsi.upc.es). The binding positions of one AP-1 and four NF-κB binding sites are indicated. **(B** and** C)** The mRNA levels of *p65/RelA* and *p50/NFKB1* were upregulated in CRF patients. The same RNA samples used in Figure 1C were applied to measure the mRNA levels of* p65* **(B)** and *p50* **(C)**. *P < 0.05.** (D** and** E)** The *c-Jun* and* c-FOS* mRNA levels were upregulated in CRF patients. The same RNA samples used in Figure 1C were applied to measure the mRNA levels of* c-Jun* **(D)** and *c-FOS* **(E)**. *P < 0.05. **(F)** The effects of knockdown of NF-κB and AP-1 subunits on the expression of *TGFB1*. Cells with knockdown of *p65*, *p50*, *c-Jun* or *c-FOS* were treated with or without 50 ng/mL recombinant IL-1β, followed by RNA isolation and qRT-PCR analyses to measure *TGFB1* mRNA levels. **P < 0.01 and *** P < 0.001. **(G)** The effects of blocking the NF-κB and AP-1 signaling pathways on the expression of *TGFB1*. Cells were primarily treated with two NF-κB signaling inhibitors, TPCA-1 and BOT-64, and two AP-1 signaling inhibitors, T-5224 and SR11302, respectively, followed by further treatment with or without 50 ng/mL IL-1β. After RNA isolation, qRT-PCR analyses were performed to measure *TGFB1* mRNA levels. **P < 0.01 and *** P < 0.001.

To explore the underlying mechanism of *TGFB1* induction in CRF patients, we primarily analyzed the TF binding sites in the promoter of *TGFB1*. We selected a 1500-bp length of the promoter and found four NF-κB sites [-189-(-198), -629-(-)638, -779-(-)788 and -881-(-)890] and one AP-1 site [-337-(-343)] (Figure 2A). The mammalian genome encodes five NF-κB members, including p65/RELA, RELB, c-REL, p50/NFKB1 and p52/NFKB2, and two AP-1 subunits, including c-Jun and c-FOS. Since *TGFB1* mRNA levels were significantly induced in CRF patients, we speculated that NF-κB and AP-1 might be also upregulated in the same samples if they could directly regulate the expression of *TGFB1*. Thus, we measured the mRNA levels of two members of NF-κB, *p65* and *p50,* and two subunits of AP-1 in CRF renal samples. Interestingly, we found that both the NF-κB and AP-1 subunits were slightly upregulated in CRF patients compared to controls (Figures 2B-2E). To further examine whether NF-κB or AP-1 was directly involved in the regulation of *TGFB1* expression, we knocked down *p65*, *p50*, *c-Jun* and *c-FOS* in a human RPTEC-TERT1 OAT3 cell line obtained from normal kidney. After verification of their successful knockdown (Supplementary Figures 1A-1D), we examined *TGFB1* mRNA levels in these cells. Our results indicated that knockdown of AP-1 subunits but not NF-κB subunits significantly repressed the expression of *TGFB1* (Figure 2F). We also used recombinant IL-1β to stimulate these knockdown cells. The results showed that the expression of *TGFB1* in sip65- and sip50-cells after IL-1β stimulation was significantly induced to a comparable level as that in control cells (Figure 2F). However, knockdown of *c-Jun* and *c-FOS* impaired the induction of *TGFB1* with IL-1β stimulation (Figure 2F), which was significantly different from that in control and sip65- and sip50-cells (Figure 2F). In addition, we also treated RPTEC-TERT1 OAT3 cells with two inhibitors of NF-κB signaling, TPCA-1 and BOT-64, and two AP-1 inhibitors, T-5224 and SR11302, respectively. Consistent with the above knockdown results, we also observed that only AP-1 inhibitors but not NF-κB inhibitors can decrease the expression of *TGFB1*, and IL-1β stimulation can only slightly induce *TGFB1* levels in cells previously treated with AP-1 inhibitors (Figure 2G). These results suggested that AP-1 was able to regulate the expression of *TGFB1 in vitro.*

### AP-1 formed a transcriptional complex with p300 and CtBP2

**Figure 3:**
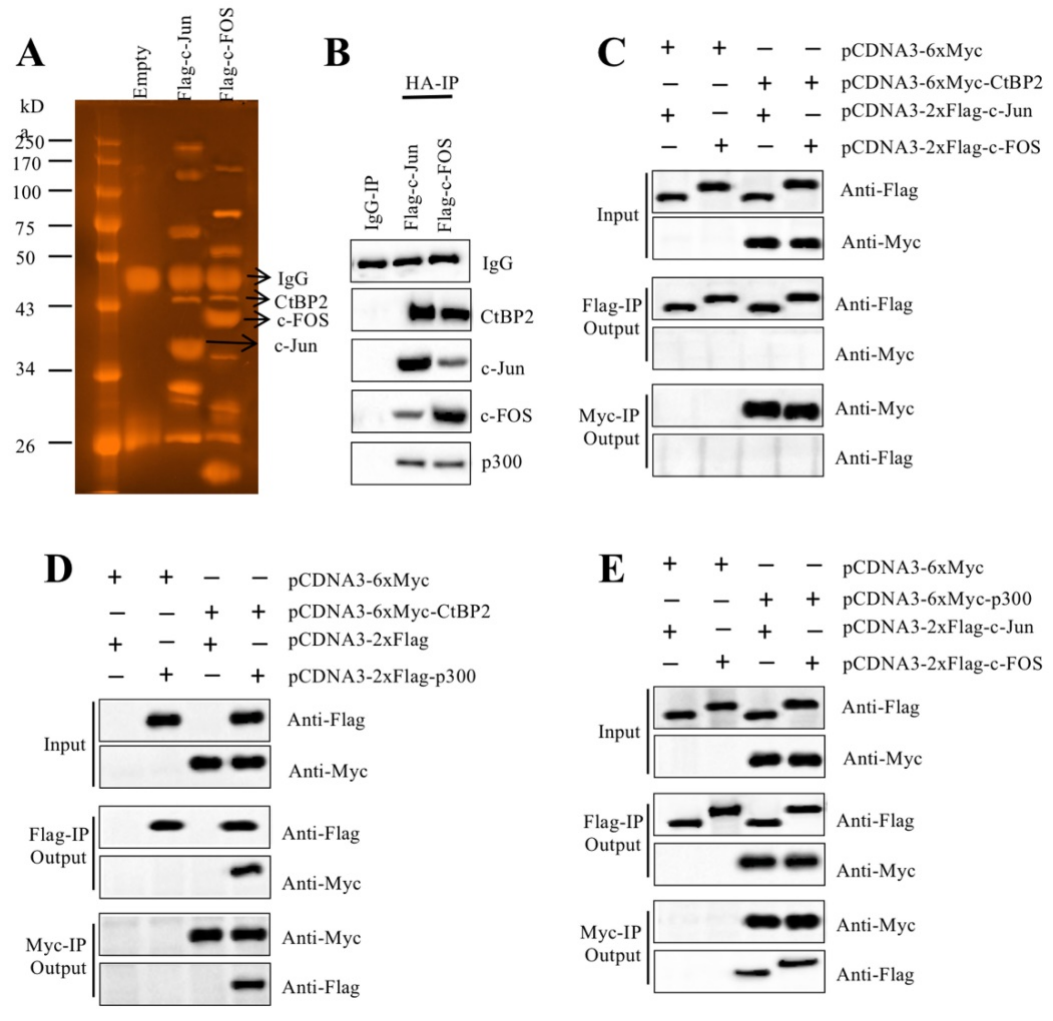
** CtBP2 formed transcriptional complexes with p300 and c-Jun or c-FOS. (A)*** In vivo* pull-down of the Flag-c-Jun- and c-FOS-associated complexes. RPTEC/TERT1 OAT3 cells were transfected with pCDNA3-2×Flag, pCDNA3-2×Flag-c-FOS or pCDNA3-2×Flag-c-Jun. After incubation for another 48 h, cells were subjected to immunoprecipitation analysis. The purified protein complexes were loaded onto SDS-PAGE gels, and protein bands were visualized using sliver staining. The IgG, c-Jun, c-FOS and CtBP2 bands are indicated. **(B)** Verification of the association of CtBP2, p300 and c-Jun or c-FOS *in vivo*. Protein samples used for mass spectrometry were applied to western blotting analyses to verify the association of CtBP2, p300 and c-Jun or c-FOS. **(C)** CtBP2 could not interact directly with c-Jun or c-FOS. The RPTEC/TERT1 OAT3 cells were transfected with the following combinations of plasmids: pCDNA3-6×Myc+pCDNA3-2×Flag-c-Jun; pCDNA3-6×Myc+pCDNA3-2×Flag-c-FOS; pCDNA3-6×Myc-CtBP2+pCDNA3-2×Flag-c-Jun; and pCDNA3-6×Myc-CtBP2+pCDNA3-2×Flag-c-FOS. After 48 h, cells were subjected to immunoprecipitation analysis using anti-Flag agarose and anti-Myc agarose, respectively. The pull-down products were used to determine protein interactions by probing with the antibodies indicated in the figures. **(D)** CtBP2 directly interacts with p300. The RPTEC/TERT1 OAT3 cells were transfected with the following combinations of plasmids: pCDNA3-6×Myc+pCDNA3-2×Flag; pCDNA3-6×Myc+pCDNA3-2×Flag-p300; pCDNA3-6×Myc-CtBP2+pCDNA3-2×Flag; and pCDNA3-6×Myc-CtBP2+pCDNA3-2×Flag-p300. After 48 h, cells were subjected to immunoprecipitation analysis using anti-Flag agarose and anti-Myc agarose, respectively. The pull-down products were used to determine protein interactions by probing with the antibodies indicated in the figures. **(E)** p300 directly interacted with c-Jun or c-FOS. The RPTEC/TERT1 OAT3 cells were transfected with the following combinations of plasmids: pCDNA3-6×Myc+pCDNA3-2×Flag-c-Jun; pCDNA3-6×Myc+pCDNA3-2×Flag-c-FOS; pCDNA3-6×Myc-CtBP2+pCDNA3-2×Flag-c-Jun; and pCDNA3-6×Myc-CtBP2+pCDNA3-2×Flag-c-FOS. After 48 h, cells were subjected to immunoprecipitation analysis using anti-Flag agarose and anti-Myc agarose, respectively. The pull-down products were used to determine protein interactions by probing with the antibodies indicated in the figures.

TFs recruit many other proteins to regulate gene expression 16, 17. To identify the members of the AP-1-associated transcriptional complex, we transfected RPTEC-TERT1 OAT3 cells with pCDNA3-2×Flag-c-FOS and pCDNA3-2×Flag-c-Jun. After immunoprecipitation with anti-Flag agarose, the Flag-c-FOS and Flag-c-Jun-associated protein complexes were applied to sliver staining (Figure 3A) followed by mass spectrometry analysis to identify proteins in these two complexes. We identified 35 proteins in the Flag-c-FOS-associated complex (Supplementary Table 4) and 29 proteins in the Flag-c-Jun-associated complex (Supplementary Table 5). Comparing these two protein lists, we found that p300 and CtBP2 overlapped (Supplementary Table 4 and 5). This result, together with previous publications 23, 30, 31, suggested that these two proteins had high possibilities of being recruited by c-Jun and c-FOS, thereby forming two transcriptional complexes. To verify this hypothesis, we primarily examined the *in vivo* association of CtBP2, p300 and AP-1 subunits. Accordingly, we determined the existence of CtBP2 and p300 in the immunoprecipitated protein materials used for mass spectrometry analysis. The results showed that both CtBP2 and p300 can be detected in both Flag-c-FOS and Flag-c-Jun-associated complexes (Figure 3B). Then, we performed Co-IP assays to examine their direct interactions. To determine if CtBP2 can directly interact with c-FOS and c-Jun, we cotransfected the following combinations of plasmids including pCDNA3-6×Myc+pCDNA3-2×Flag-c-Jun; pCDNA3-6×Myc+pCDNA3-2×Flag-c-FOS; pCDNA3-6×Myc-CtBP2+pCDNA3-2×Flag-c-Jun; and pCDNA3-6×Myc-CtBP2+pCDNA3-2×Flag-c-FOS into RPTEC-TERT1 OAT3 cells. The Co-IP results suggested that CtBP2 cannot directly interact with c-Jun or c-FOS (Figure 3C). To determine if CtBP2 directly interacted with p300, we cotransfected the following combinations of plasmids including pCDNA3-6×Myc+pCDNA3-2×Flag; pCDNA3-6×Myc+pCDNA3-2×Flag-p300; pCDNA3-6×Myc-CtBP2+pCDNA3-2×Flag; and pCDNA3-6×Myc-CtBP2 + pCDNA3-2×Flag-p300 into RPTEC-TERT1 OAT3 cells. The Co-IP results suggested that CtBP2 could directly interact p300 (Figure 3D). Following this, we determined the direct interactions of p300-c-Jun and p300-c-FOS using cells transfected with pCDNA3-6×Myc+pCDNA3-2×Flag-c-Jun; pCDNA3-6×Myc+pCDNA3-2×Flag-c-FOS; pCDNA3-6×Myc-p300+pCDNA3-2×Flag-c-Jun; and pCDNA3-6×Myc-p300+pCDNA3-2×Flag-c-FOS. The results showed that p300 directly interact with c-Jun and c-FOS (Figure 3E). Thus, we concluded that AP-1 subunits directly interacted with p300, which further recruited CtBP2 to form the AP1-p300-CtBP2 transcriptional complex.

### The CtBP2-p300-AP1 complex was upregulated in CRF patients

**Figure 4:**
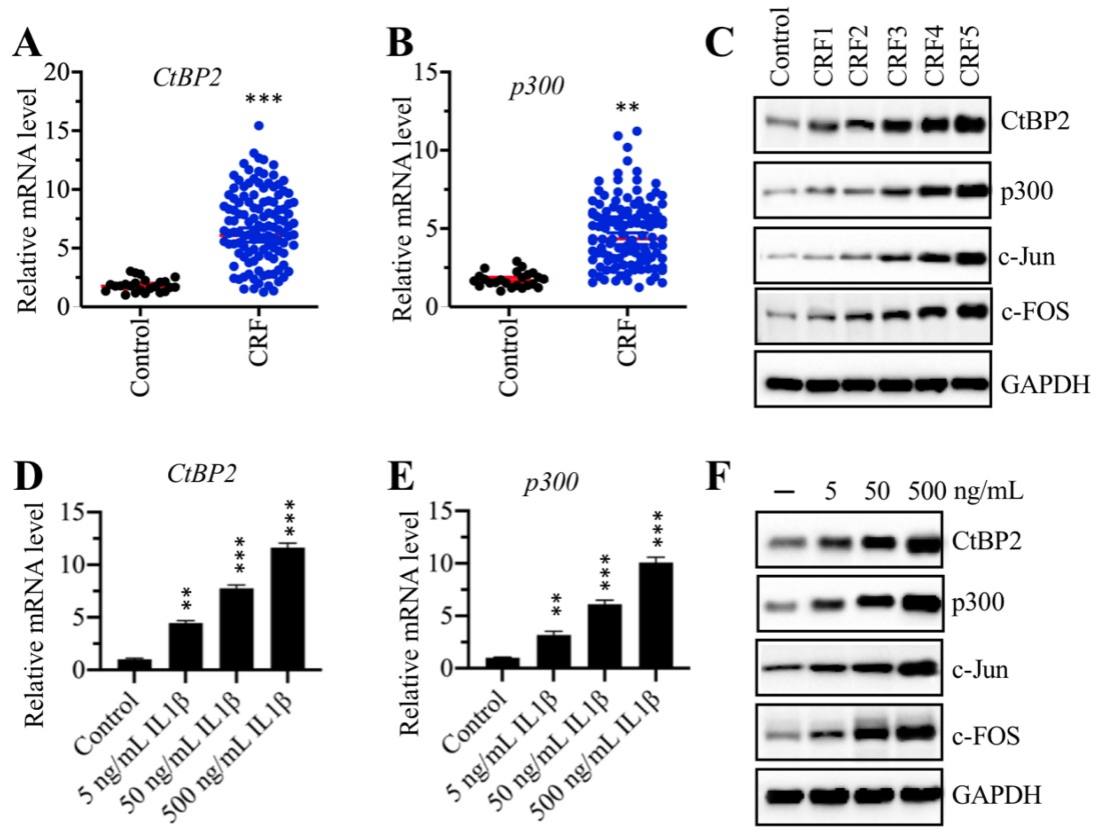
** The CtBP2-p300-AP1 transcriptional complexes were upregulated in CRF patients. (A)** The *CtBP2* mRNA level was elevated in CRF patients. The RNA samples used in Figure 1C were subjected to measurement of the mRNA level of *CtBP2*. ***P < 0.001. **(B)** The *p300* mRNA level was elevated in CRF patients. The RNA samples used in Figure 1C were subjected to measurement of the mRNA level of *p300*. **P < 0.01. **(C)** The protein levels of CtBP2-p300-AP1 components increased with the severity of CRF stage. The protein samples used in Figure 1E were used to determine the levels of CtBP2, p300, c-Jun and c-FOS. GAPDH was used as a loading control. **(D)** IL-1β stimulation induced the expression of *CtBP2*. Cells were treated with different concentrations of IL-1β including 0, 5, 50 and 500 ng/mL for 2 h, followed by RNA isolation and qRT-PCR analyses to examine *CtBP2* mRNA levels. **P < 0.01 and ***P < 0.001.** (E)** IL-1β stimulation induced the expression of *p300*. RNA samples used in (D) were subjected to qRT-PCR analyses to examine the *p300* mRNA level. **P < 0.01 and ***P < 0.001.** (F)** The protein levels of CtBP2-p300-AP1 components were increased with IL-1β stimulation. Cells used in (D) were subjected to protein isolation to determine levels of CtBP2, p300, c-Jun and c-FOS. GAPDH was used as a loading control.

Our above results indicated that the mRNA levels of *TGFB1*, *c-Jun* and *c-FOS* were upregulated in CRF biopsies. We next sought to examine the expression of *CtBP2* and *p300* in the same biopsies. The results showed that both *CtBP2* and *p300* mRNA levels were significantly increased in CRF patients compared to controls (Figures 4A and 4B). In addition, we also measured their protein levels in a control biopsy and five CRF biopsies at different stages.

Our results indicated that both CtBP2 and p300, as well as c-FOS and c-Jun, gradually increased with the severity of the CRF stage (Figure 4C and Supplementary Figure 2A). Recombinant IL-1β stimulation could result in the induction of *TGFB1*; thus, we next evaluated the effect of IL-1β stimulation on the mRNA and protein levels of CtBP2 and p300. Accordingly, we treated RPTEC-TERT1 OAT3 cells with different concentrations (0, 5, 50 and 500 ng/mL) of IL-1β. The qRT-PCR analysis results showed that both *CtBP2* and *p300* mRNA levels were significantly increased after IL-1β stimulation in a dose-dependent manner (Figures 4D and 4E). Consistent with their mRNA levels, we also observed that the protein levels of CtBP2, p300, c-Jun and c-FOS were induced with IL-1β treatment, and their inductions were dependent on the dose of IL-1β (Figure 4D and Supplementary Figure 2B).

### CtBP2 served as a coactivator in the regulation of *TGFB1* expression

**Figure 5:**
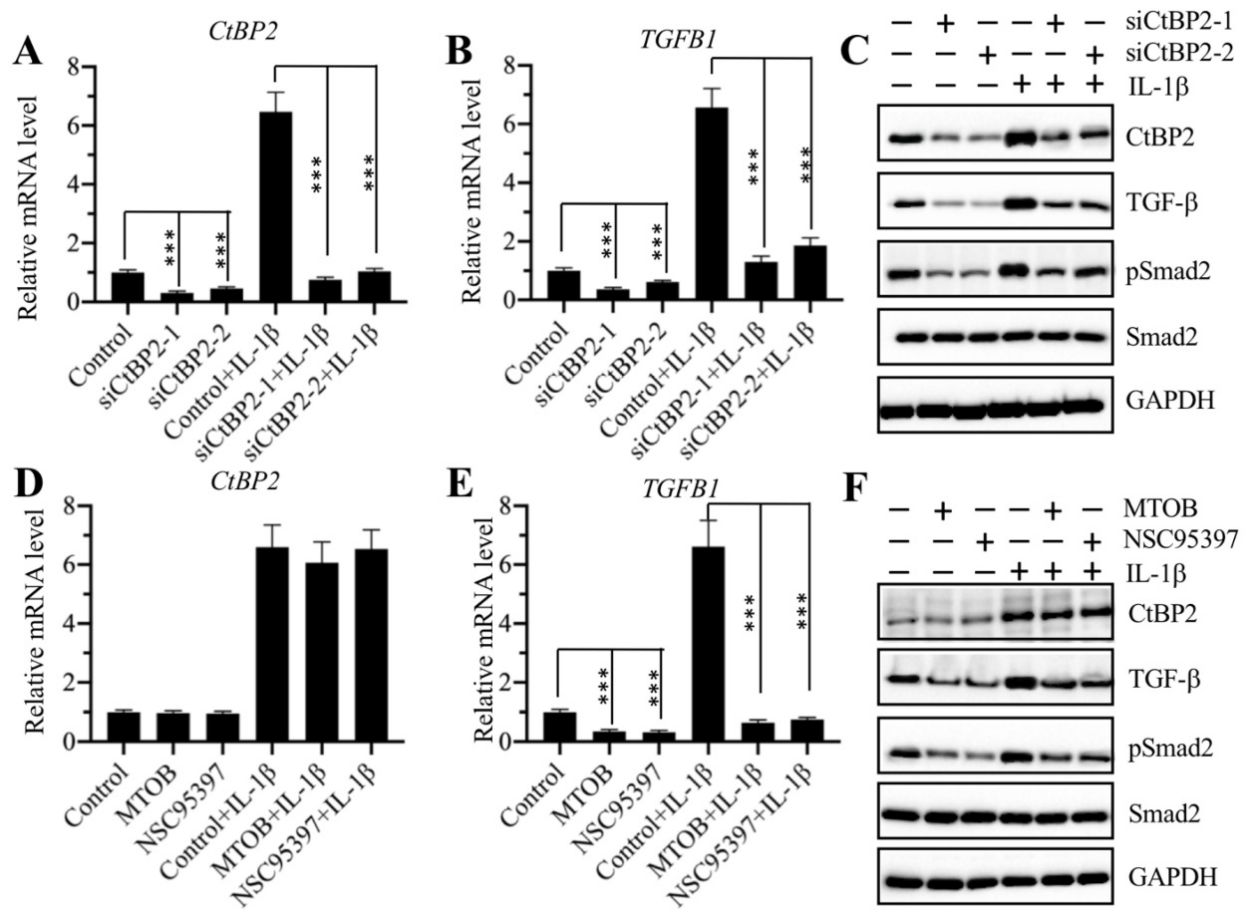
** Knockdown of *CtBP2* caused the repression of *TGFB1*. (A)** The* CtBP2* mRNA level. The RPTEC/TERT1 OAT3 cells were transfected with two independent siRNAs of *CtBP2*. After incubation for another 48 h, cells were further treated with or without 50 ng/mL IL-1β for 2 h, followed by RNA isolation and qRT-PCR analysis to measure *CtBP2* mRNA levels. ***P < 0.001. **(B)** The *TGFB1* mRNA level. RNA samples used in (A) were applied to qRT-PCR analysis to measure *TGFB1* mRNA levels. **P < 0.01 and ***P < 0.001. **(C)** Knockdown of *CtBP2* inhibited TGF-β signaling. Cells used in (A) were subjected to protein isolation and western blotting to examine the protein levels of CtBP2, TGF-β, pSmad2, Smad2 and GAPDH. **(D)** Treatments with CtBP inhibitors could not change the mRNA level of *CtBP2*. The RPTEC/TERT1 OAT3 cells were treated with 5 μM MTOB or NSC95397 for 4 h. Cells were then further treated with or without 50 ng/mL IL-1β for 2 h. The resulting cells were subjected to RNA isolation and qRT-PCR analysis to measure *CtBP2* mRNA levels. **(E)** Treatments with CtBP inhibitors significantly repressed *TGFB1* mRNA levels. RNA samples used in (D) were applied to qRT-PCR analysis to measure *TGFB1* mRNA levels. ***P < 0.001. **(F)** Treatments with CtBP inhibitors inhibited TGF-β signaling. Cells used in (D) were subjected to protein isolation and western blotting to examine the protein levels of CtBP2, TGF-β, pSmad2, Smad2 and GAPDH.

The same expression patterns of the *CtBP2*, *p300*, and *AP-1* subunits and *TGFB1* in CRF biopsies and in IL-1β-treated cells implied that CtBP2 might not serve as a corepressor but instead as a coactivator in the regulation of *TGFB1* expression. To verify this hypothesis, we knocked down *CtBP2* with two specific siRNAs in RPTEC-TERT1 OAT3 cells to generate siCtBP2-1 and siCtBP2-2 cells, followed by treatment with or without recombinant IL-1β. Αfter verifying the successful knockdown of *CtBP2* and induction by IL-1β (Figure 5A), we evaluated the expression of *TGFB1*. The results showed that knockdown of *CtBP2* dramatically decreased the expression of *TGFB1*, and the induction of *TGFB1* in the treatment of IL-1β was also dependent on CtBP2 because IL-1β stimulation only slightly increased *TGFB1* expression in *CtBP2*-knockdown cells (Figure 5B). Since changes in *CtBP2* expression could affect *TGFB1* expression, it should also affect TGF-β downstream signaling. For this purpose, we also determined the protein levels of CtBP2, TGF-β, pSmad2 and Smad2 in *CtBP2*-knockdown cells as well as in cells treated with or without IL-1β. Consistent with their mRNA levels, we also found a significant decrease in TGF-β protein levels after *CtBP2* knockdown, and IL-1β only slightly induced the expression of TGF-β (Figure 5C and Supplementary Figure 3A). As expected, *CtBP2* knockdown caused a block in TGF-β downstream signaling because we observed a significant decrease in the pSmad2 level (Figure 5C and Supplementary Figure 3A). To further confirm our observation, we also treated RPTEC-TERT1 OAT3 cells with two CtBP inhibitors including MTOB and NSC95397, which have been reported to block CtBP function and CtBP-mediated signaling in multiple publications 20. Similarly, we also examined the mRNA levels of *CtBP2* and *TGFB1* with the treatment of these two inhibitors. Our results showed that both MTOB and NSC95397 treatments could not change *CtBP2* mRNA levels; however, they significantly decreased the expression of *TGFB1* (Figures 5D and 5E). In the treatments with MTOB or NSC95397, IL-1β stimulation still markedly induced the expression of *CtBP2*, but not *TGFB1* (Figures 5D and 5E). Similarly, we also examined the protein levels of CtBP2, TGF-β, pSmad2 and Smad2 in cells treated with MTOB and NSC95397, as well as in the condition of IL-1β stimulation. Consistently, we found that both MTOB and NSC95397 treatments significantly decreased TGF-β and pSmad2 levels, and IL-1β stimulation only slightly increased their expression (Figure 5F and Supplementary Figure 3B). These results supported that CtBP2 functioned as a coactivator in the regulation of *TGFB1* expression and TGF-β downstream signaling.

### The CtBP2-p300-AP1 transcriptional complex specifically bound to the promoter of *TGFB1*

**Figure 6:**
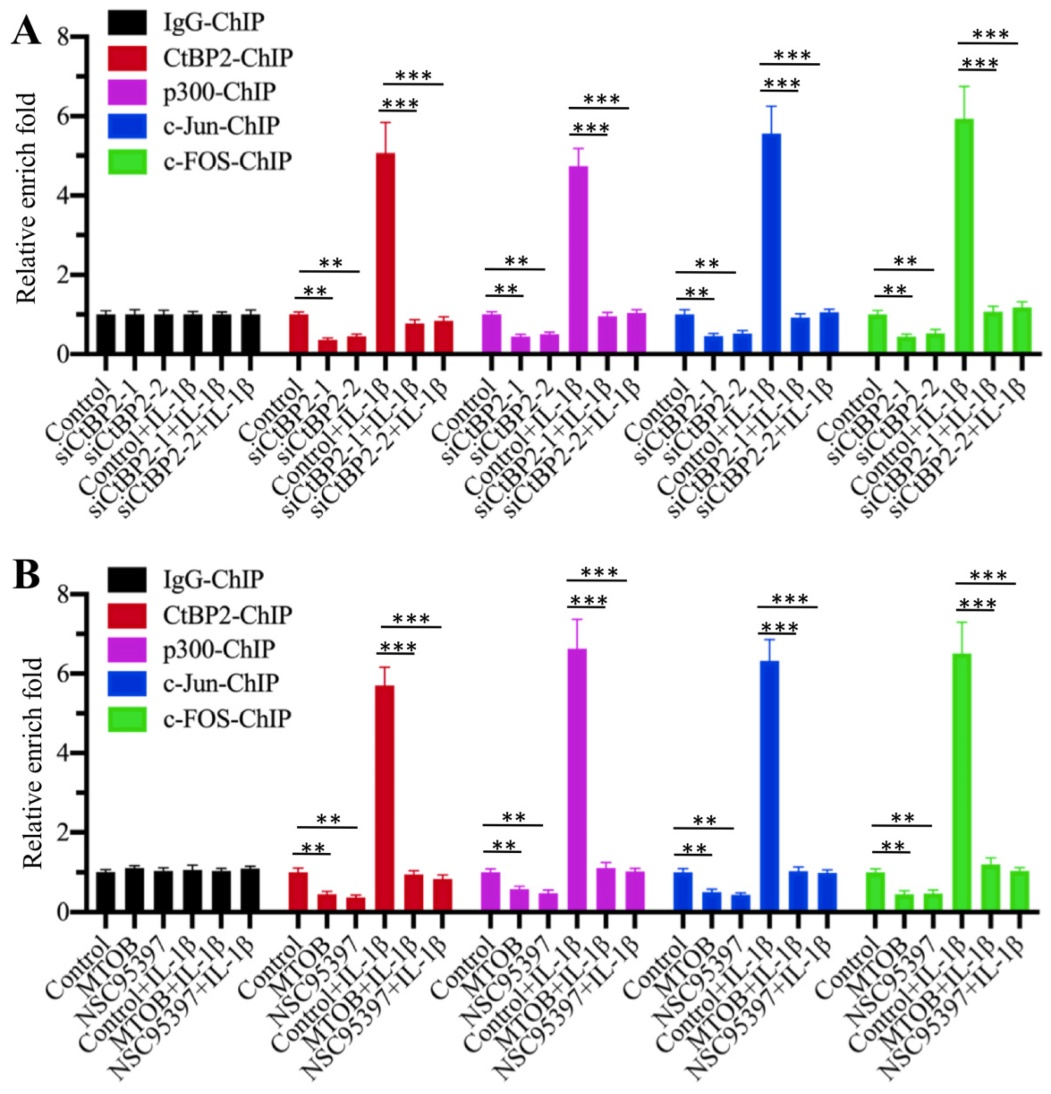
** Knockdown or blockage of CtBP2 decreased the occupancies of p300 and AP-1 in the promoter of *TGFB1*. (A)** Knockdown of *CtBP2* decreased the occupancies of p300 and AP-1 in the promoter of *TGFB1*. The RPTEC/TERT1 OAT3 cells were transfected with two independent siRNAs of *CtBP2*. After incubation for another 48 h, cells were further treated with or without 50 ng/mL IL-1β for 2 h, followed by ChIP assays using anti-CtBP2, anti-p300, anti-c-Jun and anti-c-FOS to determine their occupancies in the promoter of *TGFB1*. **P < 0.01 and ***P < 0.001.** (B)** Blockage of CtBP2 decreased the occupancies of p300 and AP-1 in the promoter of *TGFB1*. The human RPTEC/TERT1 OAT3 cells were treated with 5 μM MTOB or NSC95397 for 4 h. Cells were then further treated with or without 50 ng/mL IL-1β for 2 h. The resulting cells were subjected to ChIP assays using anti-CtBP2, anti-p300, anti-c-Jun and anti-c-FOS to determine their occupancies in the promoter of *TGFB1*. **P < 0.01 and ***P < 0.001.

Our above results suggested that the CtBP2-p300-AP1 complex activated the expression of *TGFB1*. To further verify the activation role of CtBP2 and determine whether this activation occurred through the direct binding of the CtBP2-p300-AP1 complex to the promoter of *TGFB1*, we primarily evaluated whether CtBP2 knockdown affected the occupancies of p300, c-Jun and c-FOS in the promoter of *TGFB1*. Accordingly, we performed ChIP assays in *CtBP2*-knockdown cells using anti-CtBP2, anti-p300, anti-c-Jun and anti-c-FOS for immunoprecipitation. The results showed that the occupancies of p300, c-Jun and c-FOS in the promoter of *TGFB1* were significantly decreased with *CtBP2* knockdown (Figure 6A). IL-1β stimulation significantly increased the occupancies of p300 (~5-fold), c-Jun (~6-fold) and c-FOS (~7-fold) in control cells, while it only slightly increased (~1-fold) the occupancies of these proteins in *CtBP2*-knockdown cells (Figure 6A). In addition, we also performed the same ChIP assays in cells treated with CtBP inhibitors. Similar to the results in *CtBP2*-knockdown cells, we also observed a significant decrease (~0.3-0.5-fold) in p300, c-Jun and c-FOS occupancies in cells treated with MTOB or NSC95397 (Figure 6B). Combined treatment with CtBP inhibitors and IL-1β stimulation also only slightly increased the occupancies of p300, c-Jun and c-FOS (Figure 6B). These results suggested that CtBP2 acted as an activator to induce the occupancy of p300-AP1 in the promoter of *TGFB1*.

## Discussion

Although TGF-β induction is recognized as a hallmark of CRF 13-15, 29, 31, little is known about whether its concentration is associated with the severity of the CRF stages. Importantly, the molecular mechanism of TGF-β induction is still unknown. To solve these two questions, we primarily measured serum TGF-β concentrations in a total of 120 CRF patients at different CRF stages and revealed that the TGF-β concentration was associated with the severity of CRF stages (Figure 1A). This result suggested that the serum TGF-β concentration might be used as a marker for the diagnosis of CRF. Following this, we used multiple approaches, such as bioinformatics, immunoprecipitation, mass spectrometry, Co-IP and ChIP assays, to identify and verify that the CtBP2-p300-AP1 complex was responsible for the upregulation of *TGFB1*. To our knowledge, this is the first identification of the activating role of CtBP2 in inflammatory diseases.

Most recently, Zhao and colleagues found that CtBP1 promotes metastasis of breast cancer through activating TGF-β signaling 32. On the other hand, they also found that CtBP1 and ZEB1 (zinc finger E-box binding homeobox 1) are downstream factors of TGF-β signaling 32. Thus, their findings reveal a positive feedback loop between CtBP1-ZEB1 and TGF-β signaling 32. Their results together with our findings suggest that CtBP associates with TFs and may activate TGF-β signaling in different biological processes. In addition to this activation, several studies have also revealed that CtBPs can form different transcriptional complexes with p300-Runx2 or HDAC1/2-IRF1 (interferon regulatory factor 1) 23, 33. In these models, CtBPs function as corepressors, although these complexes also share similar assembly mechanisms in which TFs specifically bind to gene promoters and then recruit histone modification enzymes (p300 or HDAC1/2) and CtBPs 23, 33. These results demonstrate that CtBPs can function as both coactivators and corepressors in the regulation of gene expression. However, we still do not understand the underlying mechanism that determines its activation or repression role.

Given that TGF-β is a central player in CRF, pharmacological intervention of TGF-β is considered as a potential strategy to decrease CRF progression 34. In our investigation, we found that two AP1 inhibitors, T-5224 and SR11302, and two CtBP inhibitors, MTOB and NSC95397, could impair the assembly of the CtBP2-p300-AP1 complex, thereby reducing the expression of *TGFB1* (Figures 2F and 5E). These results suggest that each component of the CtBP2-p300-AP1 complex can be developed as a therapeutic target in the treatment of CRF. In addition, some p300 inhibitors, such as C646 and garcinol, are also commercially available. We are currently evaluating the *in vivo* effects of these inhibitors in a CRF mouse model. Another critical issue is that we only found CtBP1 in the Flag-c-Jun-associated complex but not in the Flag-c-FOS-associated complex in our mass spectrometry (Supplementary Table 4 and 5). We did not examine CtBP1 mRNA and protein levels in CRF biopsies, so we cannot conclude if CtBP1 was involved in the regulation of *TGFB1* at present.

**Figure 7:**
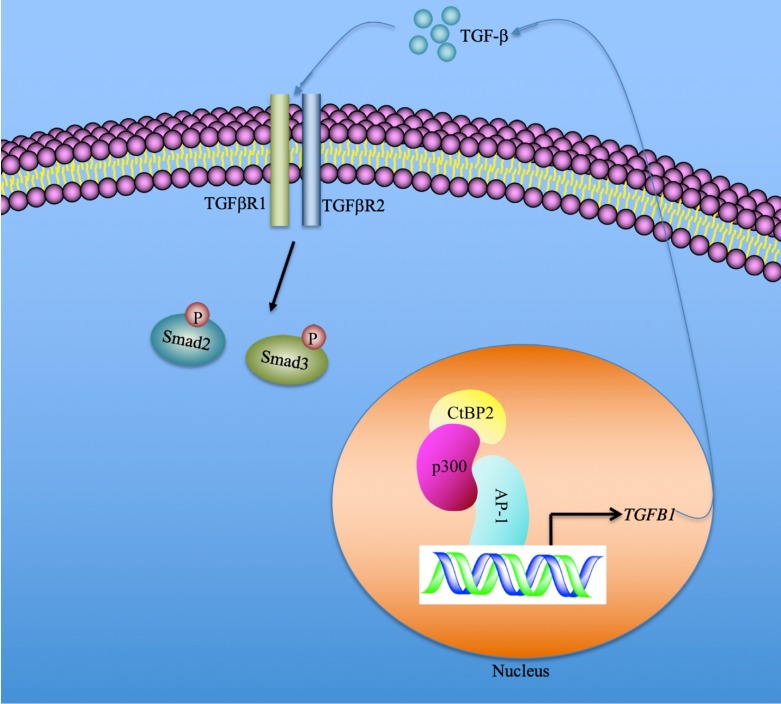
** A schematic model of the induction of *TGFB1* mediated by the CtBP2-p300-AP1 transcriptional complex.** In the nucleus, the transcription factor AP-1 specifically binds to the promoter of *TGFB1*. AP-1 further recruits histone acetyltransferase p300 to assemble a platform of AP1-p300, which further recruits CtBP2 to form a CtBP2-p300-AP1 complex. This complex activates the expression of *TGFB1* and promotes the production of mature TGF-β. The mature TGF-β is secreted to the extracellular space and functions to stimulate and to initiate TGF-β signaling, such as increasing the phosphorylation of Smad2 and Smad3.

In summary, we identified that the serum concentration of TGF-β was associated with the severity of the CRF stages. CtBP2-mediated transactivation was responsible for TGF-β induction in the pathogenesis of CRF. Our results support a model in which CtBP2-p300-AP1 transcriptional complex specifically binds to the promoter of *TGFB1* and induces its expression. The mature TGF-β is secreted into the extracellular space and functions as a stimulatory signal to activate downstream signaling, including the increase in Smad2 and Smad3 phosphorylation patterns (Figure 7). Pharmacological intervention of TGF-β signaling using inhibitors of the CtBP2-p300-AP1 complex may present new therapeutic strategies for CRF.

## Supplementary Material

Supplementary figures and tables.
