# Supplementary material for: Transforming growth factor beta (TGF-β) is activated by the CtBP2-p300-AP1 transcriptional complex in chronic renal failure
Source: Int J Biol Sci. 2020 Jan 1;16(2):204–15. doi: 10.7150/ijbs.38841 (PMC6949151; doi:10.7150/ijbs.38841)
Supplement: Supplementary file 1 — Supplementary figures and tables. [file ijbsv16p0204s1.pdf]

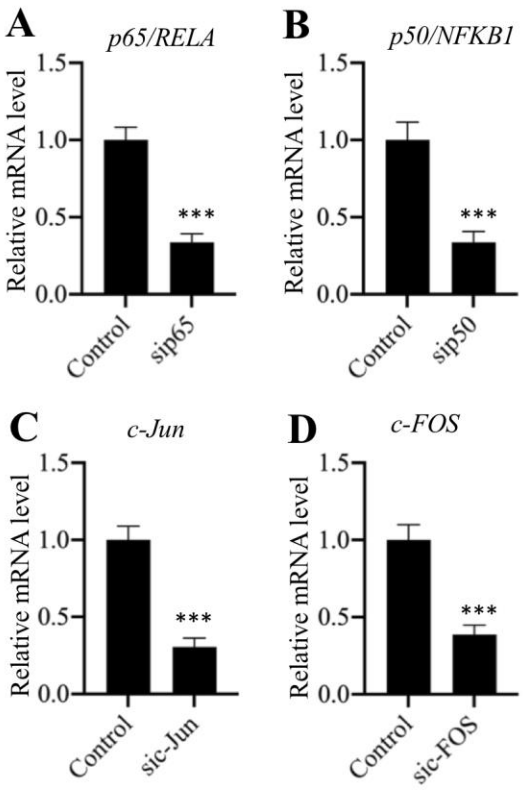

Supplementary Figure 1. The mRNA **levels of NF-κB and AP-1 subunits in their corresponding knockdown cells.**

Human RPTEC/TERT1 OAT3 cells were transfected with sip65 **(A)**, sip50 **(B)**, sic-Jun **(C)** and sic-FOS **(D)**. After incubation for another 48 h, cells were subjected to RNA isolation and qRT-PCR analyses to measure mRNA levels of p65, p50, c-Jun and c-FOS. *** $P < 0.001$.

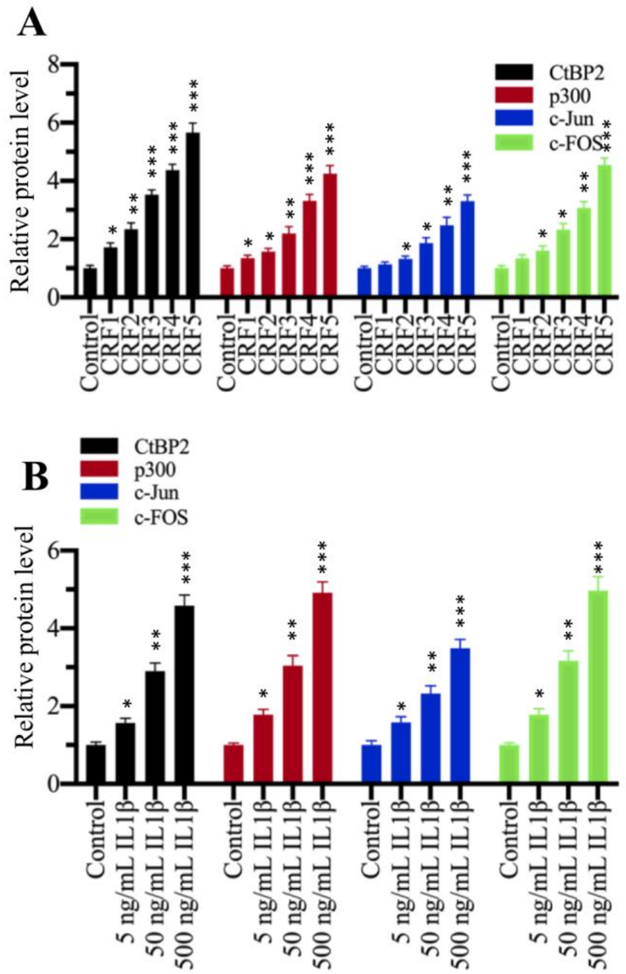

Supplementary Figure 2. The relative protein levels of CtBP2-p300-AP1 components in CRF biopsies and IL-1β-treated cells.

The protein levels of CtBP2-p300-AP1 components in Figure 4C and Figure 4F were quantified using ImageJ software. *P < 0.05, **P < 0.01 and ***P < 0.001.

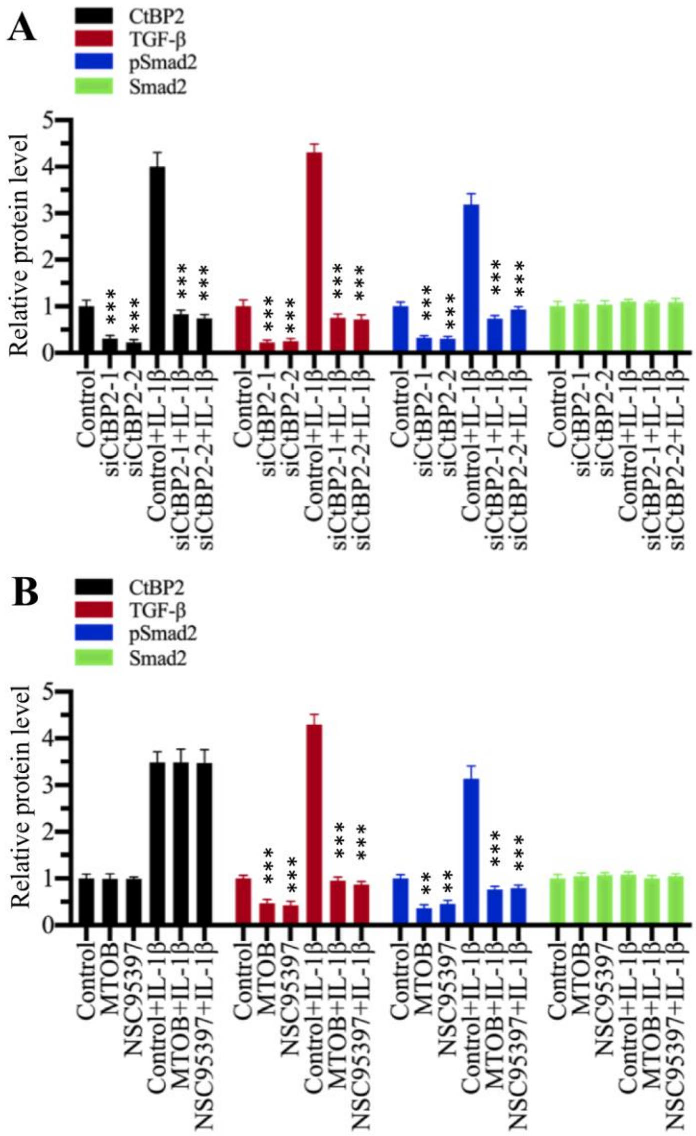

Supplementary Figure 3. The relative protein levels of TGF-β and its downstream molecules in CtBP2 knockdown and in cells treated with CtBP inhibitors.

The protein levels in Figure 5C and Figure 5F were quantified using ImageJ software.

**$P < 0.01$ and ***$P < 0.001$.

Supplementary Table-1. The basic information of NSCLC and ALI patients (n=24 in each group)

Parameter	Healthy Controls	CRF-1	CRF-2	CRF-3	CRF4	CRF5
Mean age	43.1±5.2	46.4±6.6	50.5±4.2	49.6±6.8	55.3±6.3	63.4±5.9
Gender	12M/12F	12M/12F	12M/12F	12M/12F	12M/12F	12M/12F
Average GFR	N/A	132.5±10.6	77.4±6.3	45.2±5.4	21.5±3.7	9.6±1.1

F, female; M, male.

.

Supplementary Table-2. Primers used for qRT-PCR analyses

Gene	Forward Primers	Reverse primers
TGFB1	5′-CGGAGAAGAACTGCTGCGTGC-3′	5′-GCCCACGTAGTACACGATGG-3′
p65	5′-TCCACCTCGACGCATTGCTGTGC-3′	5′-CCAGAGCTGATACCATGGCTGGAGC-3′
p50	5′-CACGAATGACAGAGGCGTGT-3′	5′-GCGCCTTGTGAAGCTGCCAGT-3′
c-Jun	5′-GAGCTGGAGCGCCTGATAATC-3′	5′-ACCGCGGGAGCCACCATGCCTG-3′
c-FOS	5′-GCTTACTCCAGGGCTGGCGTTG-3′	5′-ATCTTCTAGTTGGTCTGTCTC-3′
CtBP2	5′-AGAATCCGAAGACCCTCTGGCA-3′	5′- TCTGATATGCCCCCTTTCAA-3′
p300	5′-CAACCATCCACTACTGGAATTCG-3′	5′-TTCCGAGCATATGCAACTAGG-3′
β-Actin	5′-AGAGCTACGAGCTGCCTGAC-3	5′-AGCACTGTGTTGGCGTACAG-3′

Supplementary Table-3. Primers used for ChIP qRT-PCR analyses

Gene	Forward Primers	Reverse primers
TGFB1 promoter	5′-GGGAGGTGCTCAGTAAAGGAGA-3′	5′-AACGGAAGGAGAGTCAGGCTG-3′

Supplementary Table-4. c-FOS-associated proteins identified by mass spectrometry

Protein	Protein description	Molecular weight (kDa)	MASCOT scores
c-FOS	Fos proto-oncogene, AP-1 transcription factor subunit	48	2312
HMOX1	Heme oxygenase 1	33	2125
p300	Histone Acetyltransferase P300	264	2085
CtBP2	C-terminal binding protein 2	49	2008
CCND1	Cyclin D1	34	1964
TRIP6	Thyroid receptor-interacting protein 6	50	1943
LRP5	LDL receptor related protein 5	179	1887
BMP6	Bone morphogenetic protein 6	57	1832
ATP5PF	ATP synthase peripheral stalk subunit F6	13	1805
CARM1	Coactivator associated arginine methyltransferase 1	66	1784
KAT2B	Lysine acetyltransferase 2B	93	1749
HIPK2	Homeodomain Interacting Protein Kinase 2	131	1699
NRIP1	Nuclear receptor interacting protein 1	127	1650
PRDM16	PR/SET domain 16	140	1624
ACIN1	Apoptotic chromatin condensation inducer 1	152	1593
NLRP3	NLR family pyrin domain containing 3	118	1558
SOX2	SRY-Box 2	34	1456
OAT	Ornithine aminotransferase	49	1411
MECOM	MDS1 and EVI1 complex locus	138	1346
CAP1	Cyclase associated actin cytoskeleton regulatory protein 1	52	1288
ACTG1	Actin Gamma 1	42	1205
USP16	Ubiquitin specific protease 16	94	1195
NTRK1	Neurotrophic receptor tyrosine kinase 1	87	1112
NCOA3	Nuclear receptor coactivator 3	155	998
PPP2CB	Protein phosphatase 2 catalytic subunit beta	36	925

NFATC1	Nuclear factor of activated T cells 1	101	899
MMP9	Matrix Metallopeptidase 9	78	824
HDAC2	Histone Deacetylase 2	55	785
TFAP2A	Transcription Factor AP-2 Alpha	48	732
NR5A1	Nuclear Receptor Subfamily 5 Group A Member 1	52	690
CTLA4	Cytotoxic T-lymphocyte associated protein 4	25	667
GPS2	G Protein Pathway Suppressor 2	37	565
EMP2	Epithelial membrane protein 2	19	433
DDX23	DEAD-box helicase 23	96	324

Supplementary Table-5. **c-Jun-associated proteins identified by mass spectrometry**

**Protein**	**Protein description**	**Molecular weight (kDa)**	**MASCOT scores**
c-Jun	Jun proto-oncogene, AP-1 transcription factor subunit	36	1544
β-Actin	Actin Beta	42	1512
MAPK10	Mitogen-activated protein kinase 10	53	1447
CtBP2	C-Terminal Binding Protein 2	49	1403
JDP2	**Jun** dimerization protein 2	19	1368
MAPK3	Mitogen-activated protein kinase 3	43	1335
p300	Histone Acetyltransferase P300	264	1287
STAT3	Signal transducer and activator of transcription 3	88	1255
TRAF2	TNF receptor associated factor 2	56	1210
ITCH	Itchy E3 ubiquitin protein ligase	103	1132
DUSP1	Dual Specificity Phosphatase 1	39	1104
HIPK2	Homeodomain interacting protein kinase 2	131	1065
CSNK2A1	Casein kinase 2 alpha 1	45	1032
CDKN1B	Cyclin dependent kinase inhibitor 1B	27	998
IKBKB	Inhibitor of nuclear factor kappa B kinase subunit beta	87	932
NCK1	NCK adaptor protein 1	43	891
NOS2	Nitric oxide synthase 2	131	843
TBP	TATA-Box binding protein	38	756
CDH1	Cadherin 1	97	745
NOD2	Nucleotide binding oligomerization domain containing 2	115	712
FPGT	Fucose-1-phosphate guanylyltransferase	68	687
NOL12	Nucleolar protein 12	25	665
MAPK1	Mitogen-Activated Protein Kinase 1	41	632
WBP4	WW domain-binding protein 4	43	604
SALL1	Spalt like transcription factor	140	584
CtBP1	C-Terminal Binding Protein 1	48	521

MOCS3	Molybdenum cofactor synthesis 3	50	486
KRT7	Keratin 7	51	442
POLR2F	RNA Polymerase II Subunit F	15	376
